# Antivenom Cross-Neutralization of the Venoms of *Hydrophis schistosus* and *Hydrophis curtus*, Two Common Sea Snakes in Malaysian Waters

**DOI:** 10.3390/toxins7020572

**Published:** 2015-02-16

**Authors:** Choo Hock Tan, Nget Hong Tan, Kae Yi Tan, Kok Onn Kwong

**Affiliations:** 1 Department of Pharmacology, Faculty of Medicine, University of Malaya, Kuala Lumpur 50603, Malaysia; 2 Department of Molecular Medicine, Faculty of Medicine, University of Malaya, Kuala Lumpur 50603, Malaysia; E-Mails: tanngethong@yahoo.com.sg (N.H.T.); kytan_kae@yahoo.com (K.Y.T.); 3 Centre for Marine & Coastal Studies (CEMACS), Universiti Sains Malaysia, Penang 11800, Malaysia; E-Mail: kokonn.kwong@gmail.com

**Keywords:** sea snake, *Hydrophis schistosus*, *Enhydrina schistosa*, *Hydrophis curtus*, *Lapemis hardwickii*, antivenom, cross-neutralization, cross-reactivity

## Abstract

Sea snake envenomation is a serious occupational hazard in tropical waters. In Malaysia, the beaked sea snake (*Hydrophis schistosus*, formerly known as *Enhydrina schistosa*) and the spine-bellied sea snake (*Hydrophis curtus*, formerly known as *Lapemis curtus* or *Lapemis hardwickii*) are two commonly encountered species. Australian CSL sea snake antivenom is the definitive treatment for sea snake envenomation; it is unfortunately extremely costly locally and is not widely available or adequately stocked in local hospitals. This study investigated the cross-neutralizing potential of three regionally produced anti-cobra antivenoms against the venoms of Malaysian *H. schistosus* and *H. curtus*. All three antivenoms conferred paraspecific protection from sea snake venom lethality in mice, with potency increasing in the following order: Taiwan bivalent antivenom < Thai monocled cobra monovalent antivenom < Thai neuro polyvalent antivenom (NPAV). NPAV demonstrated cross-neutralizing potencies of 0.4 mg/vial for *H. schistosus* venom and 0.8 mg/vial for *H. curtus*, which translates to a dose of less than 20 vials of NPAV to neutralize an average amount of sea snake venom per bite (inferred from venom milking). The cross-neutralization activity was supported by ELISA cross-reactivity between NPAV and the venoms of *H. schistosus* (58.4%) and *H. curtus* (70.4%). These findings revealed the potential of NPAV as a second-line treatment for sea snake envenomation in the region. Further profiling of the cross-neutralization activity should address the antivenomic basis using purified toxin-based assays.

## 1. Introduction

Sea snake envenomation affects individuals involved in sea water activities, including swimmers, divers and particularly fishermen. Sea snake venoms are highly lethal; the envenomation can result in rapid death through neuromuscular paralysis and/or rhabdomyolysis, which leads to acute kidney injury. The wide distribution of sea snakes in the warm coastal waters makes sea snake envenomation an occupational health hazard for fisheries-related workers. In Malaysia, cases have been reported sporadically but are not uncommon [1,2]. There are more than 10 species of sea snakes in the Malaysian waters, and of these, the beaked sea snake (*Hydrophis schistosus*, previously known as *Enhydrina schistosa*) and spine-bellied sea snake (*Hydrophis curtus*, previously known as *Lapemis curtus* or *Lapemis hardwickii*) (see [3] for the latest systematics of these hydrophiid sea snakes) are the most commonly encountered species, particularly the former because it is often entangled in fishing nets and hauled in with the catch. Mishaps happen as the fishermen try to remove them from the nets [4]. These bites, in contrast to cobra and viper bites, are relatively painless and cause no remarkable local inflammation even as the toxins are insidiously absorbed and effectively distributed throughout the systemic circulation, causing rapid death if treatment is not sought in time.

To date, the only indicated specific antidote for sea snake envenomation is Australian CSL sea snake antivenom. Unfortunately, that antivenom is extremely expensive locally (costing approx. 3000 USD per vial in Malaysia, with several vials often required for effective treatment). It is also a product that demands extreme care in transport and storage (the antivenom is in a perishable liquid form, needing constant cold storage). In addition to the unpredictable occurrence of bites, the antivenom is not widely available or adequately stocked in all Malaysian hospitals. In view of the restricted market demand and commercial difficulty faced by antivenom producers globally [5], there is also concern about whether sea snake antivenom production would be sustainable and sufficient to supply other countries, including Malaysia. Hence, a practical approach to optimizing the country’s treatment protocol would be searching for an alternative (or second-line) antivenom of choice, preferably based on the extended neutralization spectrum of a heterologous antivenom, which is more commonly stocked in local hospitals. The proposed strategy could be difficult, though, as snake venom toxins are highly variable between species; for instance, although neurotoxins are known to present in the venoms of sea snakes and cobras, there are substantial differences in their subtypes and mechanisms [6]. Nevertheless, previous studies have shown that some antivenoms can confer cross-neutralization effects on specific venom toxicities of heterologous species in animal studies, presumably due to the common antigenic properties of different venoms [7,8,9]. Earlier reports by Minton [10] and Khow *et al.* [11] suggested that Thai anti-cobra antivenom (old batches, immunoglobulin form unspecified in [10], F(ab')_2_ in [11]) could neutralize the venoms of *Enhydrina schistosa* (synonymized as *H. schistosus*; unspecified source) and *Lapemis hardwickii* (synonymized as *H. curtus*; sourced from Japan Snake Institute), respectively. However, there is still a lack of systematic characterization and standard quantitation details for cross-neutralization efficacy and potency, as well as validation using the current commercially available and clinically used Thai antivenoms (produced by Queen Saobhava Memorial Institute), which are also manufactured with a revised WHO-endorsed method [12]. This study aims to investigate the cross-neutralization profile of two common Malaysian sea snake venoms by regionally produced anti-elapid antivenoms (with anti-cobra components), with mice as the experimental envenomation model.

## 2. Experimental Section

### 2.1. Venoms and Antivenoms

Venoms were milked individually from adult *H. schistosus* and *H. curtus* collected from the Northwestern waters of Peninsular Malaya by one of the authors (CHT) into sterile polystyrene containers and lyophilized later. The venoms of two sea kraits (*Laticauda laticaudata* and *Laticauda semifasciata*) used for lethality comparison were obtained from Miami Serpentarium (Punta Gorda, FL, USA), and the venoms of *Naja kaouthia* and *Calloselasma rhodostoma* used in ELISA were obtained from Latoxan (Rosans, France). The antivenoms used were as follows: Taiwan bivalent antivenom for *Naja atra* and *Bungarus multicinctus* (batch: FN10001, expiry: 31 March 2016, manufacturer: Taiwan Central for Disease Control, Taipei, Taiwan); Thai monovalent antivenom for *Naja kaouthia* (batch: NK00310, expiry: 9 August 2015) and neuro polyvalent antivenom for *Naja kaouthia*, *Ophiophagus hannah*, *Bungarus candidus* and *B. fasciatus* (batch: NP00109; expiry: 5 October 2014, manufacturer: Queen Saovabha Memorial Institute, Bangkok, Thailand). The antivenoms were purified equine F(ab')_2_ products and weighed 0.9–1.0 g each vial. They were diluted in 10 mL saline per vial according to the manufacturers’ recommendations, to a concentration of 90–100 mg/mL.

### 2.2. Animals Use and Supply

Mice used in this study were of albino ICR strain, 4–5 weeks old, male, weighing 20–25 g, and were supplied by the Animal Experimental Unit, University of Malaya. The protocol of experimental animal use in this study was based on the guidelines given by CIOMS [13] and the use of animals was approved by the Institutional Animal Care and Use Committee of the University of Malaya (Ref: 2014-09-11/PHAR/R/TCH).

### 2.3. Lethality Study

Sea snake venoms were administered at a total volume of 100 μL via intravenous (via tail caudal vein), intramuscular (via quadriceps) or subcutaneous (via loose skin over the neck) route into albino ICR strain mice (20–25 g) at various doses (*n* = 4 per dose). The survival ratio for mice at each dose was recorded after 48 h of observation, in which the mice were given full access to food and water *ad libitum*. Median lethal doses of the venoms were determined for the different routes of administration using the Finney’s probit analysis method [14] (see Section 2.6).

### 2.4. Neutralization Study

#### 2.4.1. Preincubation Neutralization

Neutralization of lethality was conducted as described by Tan *et al.* [9]. A lethal challenge dose constituting 2.5 or 5.0 LD_50_ of sea snake venom was preincubated at 37 °C with various dilutions of antivenom in normal saline for a total volume of 200 µL. The mixture was then centrifuged at ×10,000 *g*, and the supernatant was injected into the caudal vein of the mice (*n* = 4 per dose of antivenom). The number of mice that survived after 48 h was recorded for antivenom efficacy and potency estimations (see Section 2.6).

#### 2.4.2. Challenge-Rescue Neutralization

Mice were subcutaneously envenomed with 2.5 LD_50_ of sea snake venom. Based on the result of the preincubation neutralization study (Section 2.4.1), the antivenom with the highest efficacy was injected intravenously into the experimentally envenomed mice (at different dilutions) via the caudal vein 10 min later. The number of mice that survived after 48 h was recorded for antivenom efficacy and potency estimations (see Section 2.6).

### 2.5. Immunological Cross-Reactivity Study

Immunological cross-reactivities between sea snake venoms and antivenom were examined with an indirect enzyme-linked immunosorbent assay modified from that used by Tan *et al.* [15]. The venoms of *Naja kaouthia* and *Calloselasma rhodostoma* were used for comparison with the sea snake venoms. In brief, immunoplate wells were precoated overnight with 5 ng of venom antigens (*H. schistosus*, *H. curtus*, *Calloselasma rhodostoma*, *Naja kaouthia*) at 4 °C. The plate was then flicked dry and rinsed five times with Tris-buffered saline with 0.5% Tween^®^20 (TBST). One hundred microliters of appropriately diluted antivenom (1:6000) was added to each antigen-coated well, followed by incubation for 1 h at room temperature. After washing the plate five times with TBST, 100 µL of appropriately diluted horseradish peroxidase-conjugated antihorse-IgG (Jackson ImmunoResearch Inc., West Grove, PA, USA) in TBST (1:8000) was added to the well and incubated for another hour at room temperature. The excess components were removed by washing five times with TBST. A hundred microliters of freshly prepared substrate solution (0.5 mg/mL o-phenylenediamine and 0.003% hydrogen peroxide in 0.1 M citrate-phosphate buffer, pH 5.0) was added per well. The enzymatic reaction was allowed to take place in the dark for 15 min at room temperature. The reaction was subsequently terminated by adding 50 µL of 12.5% sulfuric acid, and the absorbance at 492 nm was read against the blank using an ELISA reader (SUNRISE-TECAN Type Touch Screen F039300, Tecan, Männedorf, Switzerland). All experiments were performed in triplicate.

### 2.6. Statistical Analysis

Antivenom effectiveness was expressed as a median effective dose (ED_50_, the amount of reconstituted antivenom in µL that produces 50% survival in the animals tested), effective dose ratio (ER_50_, the amount of venom neutralized per mL of antivenom at which 50% of envenomed mice survived), as well as “potency” (P, the amount of venom that is completely neutralized by a unit volume of antivenom, calculated according to Morais *et al.* [16]). The neutralization potency (P) is theoretically unaffected by the challenge dose and also serves as an indicator for comparing “neutralizing capability” between different antivenoms for a specific test venom. The median lethal dose (LD_50_), median effective dose (ED_50_), effective dose ratio (ER_50_) and the 95% confidence intervals (C.I.) were calculated using the probit analysis method used by Finney (1952) with BioStat 2009 analysis software (AnalystSoft Inc., Vancouver, Canada). The statistical analysis for the ELISA assay was conducted using SPSS (Version 18.0, SPSS Inc., Chicago, IL, USA). The data (expressed as mean ± S.D.) were analyzed using one-way ANOVA, with Tukey’s *post hoc* multiple-comparison test, with *p* < 0.05 as the significant threshold.

## 3. Results and Discussion

All four sea snake venoms exhibited highly potent lethality in mice (Table 1), especially for the two common species in Malaysia: Both *H. schistosus* and *H. curtus* venoms possess LD_50_ < 0.1 µg/g. This indicates that the bites from these species, though they appear to be under-reported, should be taken very seriously by local health authorities. There are no significant differences (*p* > 0.05) noted in LD_50_ values between the three routes for envenoming (intravenous, intramuscular and subcutaneous), indicating that the sea snake venoms share a near-complete systemic absorption from the subcutaneous site of a snake bite. This is likely because the principal lethal toxins of sea snake venoms consist mainly of low molecular mass toxins (α-neurotoxins and phospholipases A_2_) [17] that are able to cross barrier membranes more effectively (better absorption) like cobra venom toxins [18]. Additionally, in sea snake bites, there is very minimal interaction between the local tissue and toxins (virtually no local tissue inflammation or necrosis) to retain the venom *in situ* [4]. This is in contrast to some Viperidae venoms which can show exceptionally low systemic bioavailability from a non-vascular injection site [19].

**Table 1:** Comparison of median lethal doses (LD_50_) of four sea snake venoms via different routes of administration into mice.

Venom	LD_50_* i.v.* (µg/g)	LD_50_* i.m.* (µg/g)	LD_50_* s.c.* (µg/g)
*Hydrophis schistosus (Malaysia)*	0.07 (0.05–0.09)	0.10 (0.08–0.12)	0.09 (0.06–0.14)
*Hydrophis curtus (Malaysia)*	0.11 (0.07–0.17)	0.18 (0.12–0.27)	0.18 (0.17–0.18)
*Laticauda laticaudata*	0.10 (0.08–0.12)	-	-
*Laticauda semifasciata*	0.22 (0.15–0.34)	-	-

i.v.: intravenous; i.m.: intramuscular; s.c.: subcutaneous; -: not determined. Units expressed as µg reconstituted venom per g mouse body weight.

**Figure 1:**
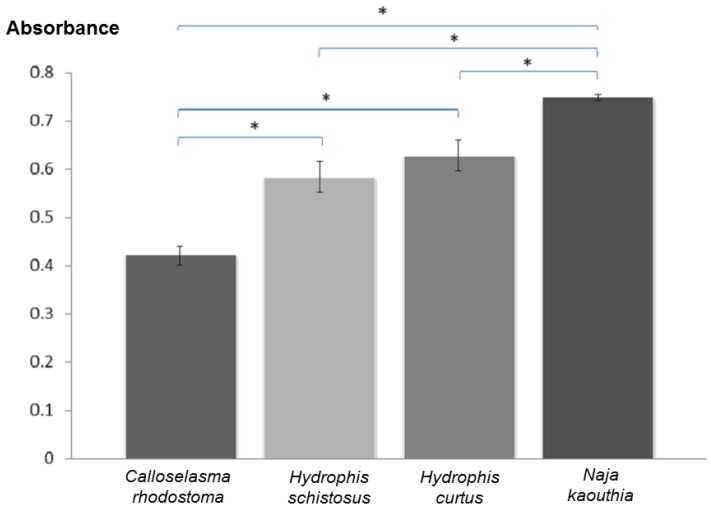
Absorbance values from indirect ELISA indicating cross-reactivities between neuro polyvalent antivenom (NPAV) with venoms from *Calloselasma rhodostoma*, *Hydrophis schistosus*, *Hydrophis curtus* and *Naja kaouthia*. Values were expressed as mean of triplicates with standard deviation. Asterisks indicate significant difference between means (*p* < 0.05).

Since the neurotoxins (NTX) in *H. schistosus* venom are typically the short-chain type, cross-neutralization was first investigated using the Taiwan bivalent antivenom (TBAV), as *Naja atra* venom, one of the venoms used to raise the antivenom, consists mainly of short-NTX [20]. Using the antivenom-venom preincubation method, TBAV was found to be only weakly effective, with potency values of 0.016 mg/mL and 0.019 mg/mL for cross-neutralizing the venoms of *H. schistosus* and *H. curtus*, respectively, in mice (Table 2). The Thai *Naja kaouthia* monovalent antivenom (NKMAV) appeared to be more effective than TBAV in this regard (Table 2), with potency values approximately twice that of TBAV, even though Thai cobra venom is known to consist mainly of long α-neurotoxins. These findings suggest that NKMAV may cross-react more substantially with the sea snake venoms. This supports an earlier report that Thai cobra long NTX-specific F(ab')_2_ cross-reacted with *H. curtus* venom [11], although the major NTX in sea snake venom is of the short NTX subtype [21]. The new polyvalent antivenom (neuro polyvalent antivenom, NPAV) produced by QSMI, which targets neurotoxic envenomation, showed a distinctively higher neutralizing potency, approximately twice that of NKMAV and four times that of TBAV, at doses standardized according to the respective guidelines for clinical use. Cross-neutralization by NPAV was also observed for sea krait (*Laticauda* sp.) venoms, which mainly comprise NTX of the short-chain type called erabutoxins [22]. In comparison with NKMAV, the apparently higher potency observed in NPAV in cross-neutralizing *H. schistosus* and *H. curtus* venoms could be due to synergistic cross-reacting effects from the polyvalent F(ab')_2_ against toxin components originating from other elapids, in particular possible neutralization of sea snake basic PLA_2_ in addition to the neurotoxins. A previous study [23] demonstrated that prior administration (10 min) of CSL tiger snake antivenom into the chick biventer cervicis nerve-muscle preparation was able to attenuate the neuromuscular blockade effect of the venoms of several sea snakes, except that produced by *E. schistosa* (synonymized as *H. schistosus*). The current *in vivo* study indicated that the antigenic properties of *H. schistosus* venom may be closer to that of the *Naja* cobra than the tiger snake. The cross-neutralization phenomenon is also supported by the immunological cross-reactivity result, where ELISA cross-reactivities between NPAV with *H. schistosus* and *H. curtus* venoms were shown to be 58.4% and 70.4% against blank, respectively (Figure 1). Comparatively, the ELISA reactivity of NPAV with the homologous *N. kaouthia* venom was high (103.3% against blank). The data indicate that the sea snake toxins have some degree of binding avidity toward NPAV, although the ELISA cross-reactivity values are not necessarily congruent or in proportion with the neutralizing potency against the different venoms. In contrast, the absorbance value for *C. rhodostoma* venom (14.4% against blank) is remarkably low, representing non-specific binding between venom proteins and NPAV without effective neutralizing activity.

The cross-neutralizing effectiveness of NPAV was further tested in an experimental envenomation model, where different doses of NPAV were administered intravenously into mice that were subcutaneously pre-envenomed with 2.5 LD_50_ of *H. schistosus* or *H. curtus* venom. NPAV was proven effective *in vivo* for neutralizing both Malaysian sea snake venoms in the experimental envenomation model (Table 2). From the experience of venom milking (using the method of induced biting through a film), we estimate the average *H. schistosus* venom yield per bite to be 6.1 ± 3.7 mg (*n* = 22, range 2–12 mg, adult snake > 1 m) and average *H. curtus* venom amount per bite to be approximately 1 mg (*n* = 3, range 0.7–1.2 mg, adult snakes of approximately 1 m). Based on the potencies (Table 2) translated into 0.4 mg/vial and 0.8 mg/vial of NPAV for *H. schistosus* and *H. curtus* venoms respectively, a total of 10–20 vials of NPAV may be required for neutralization of the venom injected per bite. In cobra envenoming, this is considered an acceptable amount for antivenom dosage; the initial dose is typically 10 vials, followed by additional doses as required clinically [24].

**Table 2:** Cross-neutralization by paraspecific antivenoms against the lethal effect of selected sea snake venoms in mice.

Venom	TBAV (preincubation)			NKMAV (preincubation)			NPAV (preincubation)			NPAV (challenge-rescue)		
	ED_50_ (µL)	ER_50_ (mg/mL)	P (mg/mL)	ED_50_ (µL)	ER_50_ (mg/mL)	P (mg/mL)	ED_50_ (µL)	ER_50_ (mg/mL)	P (mg/mL)	ED_50_ (µL)	ER_50_ (mg/mL)	P (mg/mL)
*Hydrophis schistosus*	141.36	0.027 (0.019–0.035)	0.016	89.89	0.045 (0.032–0.058)	0.027	100.00	0.074 (0.053–0.095)	0.059	70.68	0.070 (0.047–0.109)	0.042
*Hydrophis curtus*	200.00	0.032 (0.021–0.050)	0.019	89.89	0.070 (0.045–0.109)	0.042	125.00	0.092 (0.059–0.143)	0.074	70.68	0.143 (0.135–0.143)	0.086
*Laticauda laticaudata*	-	-	-	-	-	-	75.00	0.075 (0.060–0.090)	0.045	-	-	-
*Laticauda semifasciata*	-	-	-	-	-	-	50.00	0.253 (0.173–0.391)	0.15	-	-	-

TBAV: Taiwan bivalent antivenom (for *Naja atra* and *Bungarus multicinctus*); NKMAV: Thai *Naja kaouthia* monovalent antivenom; NPAV: Thai neuro polyvalent antivenom. ED_50_: median effective dose, defined as the volume of reconstituted antivenom that protects 50% of mice from death; ER_50_: Effective dose ratio, defined as amount of venom neutralized per mL antivenom at which 50% of envenomed mice survived; P: Potency, defined as amount of venom neutralized per mL antivenom in total (presumably 100% survival conferred) [16]. Parentheses indicate the 95% C.I.; -: not determined. All venom doses used in neutralization study = 2.5 LD_50_, except in pre-incubation method for NPAV where 5 LD_50_ of *H. schistosus* and *H. curtus* venoms were used. The venom challenge doses (2.5 or 5 LD_50_) were all higher than the LD_100_ values of the corresponding venoms (1.2–1.6 LD_50_).

## 4. Conclusions

The lethality of sea snake venoms does not vary significantly among routes of venom administration in mice, presumably due to effective absorption of principal toxins which are small in molecular size, such as neurotoxins and phospholipase A_2_. The anti-elapid antivenoms examined, especially NPAV, confer paraspecific neutralization against the lethal effect of sea snake venoms in mice. The potential of NPAV as a second-line treatment for sea snake envenomation, however, needs to be further substantiated with an antivenomic approach. The cross-neutralization effect can be profiled against purified sea snake principal toxins such as neurotoxins and phospholipases A_2_. Neurotoxins lead to flaccid paralysis while phospholipases A_2_ cause generalized myotoxicity [25,26]; the immunological interaction of NPAV with these toxins should be explored further for a better understanding of the paraspecific protective mechanism of NPAV against sea snake venoms.
